# Serum Copeptin, Pentraxin 3, Anti-Mullerian Hormone Levels With Echocardiography and Carotid Artery Intima-Media Thickness in Adolescents With Polycystic Ovary Syndrome

**DOI:** 10.14740/jocmr2375w

**Published:** 2015-10-23

**Authors:** Mehmet Deveer, Ruya Deveer, Ozcan Basaran, Ummuhani Ozel Turkcu, Eren Akbaba, Nesat Cullu, Nilgun Turhan, Mert Kucuk, Burcu Kasap

**Affiliations:** a Department of Radiology, School of Medicine, Mugla Sitki Kocman University, Mugla, Turkey; b Department of Obstetrics and Gynecology, School of Medicine, Mugla Sitki Kocman University, Mugla, Turkey; c Department of Cardiology, School of Medicine, Mugla Sitki Kocman University, Mugla, Turkey; d Department of Medical Biochemistry, School of Medicine, Mugla Sitki Kocman University, Mugla, Turkey

**Keywords:** Copeptin, PTX3, AMH, adolescent, PCOS

## Abstract

**Background:**

The aim of the study was to investigate the presence of possible markers in the prediction of polycystic ovary syndrome (PCOS)-related metabolic alterations and cardiovascular events in adolescent PCOS cases and also to investigate the applicability of anti-Mullerian hormone (AMH) levels for the diagnosis of PCOS.

**Methods:**

In this cross-sectional study, a total of 75 non-obese women (adolescent PCOS group, n = 25; adult PCOS group, n = 25; control group, n = 25) were included. Measurements of copeptin, pentraxin 3 (PTX3), and AMH serum levels were performed.

**Results:**

Serum copeptin, PTX3 and echocardiographic indices were not significantly different in PCOS subjects and they did not have higher common carotid artery intima-media thickness (CIMT) measurement. AMH levels were significantly higher in PCOS patients. There was a positive correlation between AMH and mean ovarian volume (r = 0.58, P < 0.001) and between AMH and total testosterone level (r = 0.63, P < 0.001). In order to predict a threshold value for the diagnosis of PCOS by using AMH, the receiver operating characteristic (ROC) method was used. Area under the curve was 0.820 and cut-off point was 6.66 ng/mL for AMH with a sensitivity of 62% and specificity of 76%.

**Conclusions:**

Possible markers for PCOS-related metabolic alterations may not present in the adolescent years. Serum AMH may be useful as a diagnostic test for adolescents.

## Introduction

The polycystic ovary syndrome (PCOS) is a common endocrine disorder that affects 5-8% of women of reproductive age [1]. It usually becomes manifest during adolescence and characterized by ovulatory dysfunction, infertility and hyperandrogenism. It has lifelong implications with increased risk for metabolic syndrome, type 2 diabetes mellitus, and possibly cardiovascular disease [2]. Accurate diagnosis of PCOS during adolescence is difficult. Copeptin is a 39-aminoacid glycopeptide, which is a C-terminal part of the precursor pre-provasopressin (pre-proAVP). It is released after stress situations and is one of the mediators associated with insulin resistance, diabetes mellitus, obesity and metabolic syndrome [3, 4]. Pentraxin 3 (PTX3) is an inflammatory mediator belonging to the same family as C-reactive protein (CRP) and serum amyloid P. In response to inflammatory stimuli, it is produced by various cell types, especially in the vasculature [5]. Anti-Mullerian hormone (AMH) is a glycoprotein belonging to transforming growth factor beta (TGF-β) family. AMH levels are associated with elevated androgen levels and have been proposed as a diagnostic marker for ovarian hyperandrogenism [6], but the role of AMH in the diagnosis of PCOS is uncertain [7].

Our aim was to investigate the role of possible markers in the prediction of PCOS-related metabolic alterations and cardiovascular events and to investigate the applicability of AMH levels for the diagnosis of adolescent PCOS cases.

## Material and Methods

This cross-sectional study was conducted between May 2013 and June 2014 at Mugla Sitki Kocman University, Department of Obstetrics and Gynecology Unit. A total of 75 women (adolescent PCOS group, n = 25; adult PCOS group, n = 25; control, n = 25) were included into the study. The PCOS subjects were selected from a group of PCOS patients who were complaining of menstrual irregularity, hirsutism or infertility. The study was approved by the local ethics committee. Informed consent was obtained from all participants.

PCOS was diagnosed according to the revised consensus on diagnostic criteria (2003 revised Rotterdam criteria). After the exclusion of other etiologies, the presence of all three features below was necessary for PCOS subjects: oligo-anovulation, clinical and/or biochemical signs of hyperandrogenism, and polycystic ovaries. The ovarian volume was calculated using the formula for a prolate ellipsoid: 0.5 × length × width × thickness [8]. The mean ovarian volume was calculated by averaging the bilateral ovarian volume. Clinical hyperandrogenism was quantified by modified Ferriman-Gallwey scoring system (F-G). All participants had normal prolactin levels, and a normal renal, hepatic and thyroid function. All the patients in the study were menstruating for at least 2 years. Any history of systemic or endocrine diseases, cancer, thromboembolism, smoking, medication, and morbid obesity was considered as exclusion criteria.

The control group consisted of healthy women volunteers with regular menses and no history of hirsutism, hyperandrogenism, any systemic or endocrine disease. Six patients in the control group were older than 35 years of age (36 - 41 years). Except virgin adolescents, all patients underwent transvaginal ultrasound (GE Voluson 730 Pro).

### Measurement of copeptin, PTX3 and AMH serum levels

Blood samples were taken on days 3 - 5 of a spontaneous or progestin-induced menstrual cycle, and serum was separated and frozen at -70 °C until assayed. Copeptin concentrations were measured by enzyme-linked immunosorbent assay (ELISA) kit (Cloud-Clone Corp., Houston, TX, USA). Assay sensitivity was less than 5.7 pg/mL and inter-assay and intra-assay coefficients of variations were < 10% and < 12%, respectively. Serum PTX3 levels were measured by using Human PTX3 ELISA KIT from Boster immunoleader (Boster Biological Technology Co., Ltd, CA, USA). The sensitivity was < 10 pg/mL. AMH was measured by AMH Gen II (A79765) ELISA kit (Beckman Coulter, Inc., CA, USA) with a sensitivity of 0.08 ng/mL. Inter-assay and intra-assay coefficients of variabilities were 5.6% and 5.4% respectively.

### Echocardiographic and Doppler study

Transthoracic echocardiography was performed using a 1.5 - 3.6 MHz phased-array transducer, (Vivid S6, General Electric, Vingmed, Horten, Norway). Left ventricular mass (LVM) was calculated using the Devereux formula [9]: 0.8{1.04[([LVDd + IVSd + PWd]3 - LVDd3)]} + 0.62, where LVDd is LV end diastolic diameter, IVSd is interventricular septum diameter and PWd is LV posterior wall diameter. LV mass index (LVMi) was obtained by the following formula: LVM/body surface area. Pulsed wave tissue Doppler was also recorded. LV myocardial performance index (MPI) was determined by using equation MPI = (IRT + ICT)/ET [10], where IRT is isovolumetric relaxation time, ICT is isovolumetric contraction time, and ET is ejection time.

All echocardiographic measurements were made by a cardiologist blinded to the study protocol.

### Common carotid artery intima-media thickness (CIMT) measurement

Evaluation for bilateral CIMT was performed using high-resolution Doppler ultrasonography (Aplio 500, Toshiba, Tokyo, Japan) with a 7.5-mHz linear-array transducer. Measurements were performed at the level of the CCA bulbus 1 cm proximal from the bifurcation at the longitudinal axis [11]. Average of left and right carotid arteries was taken as one variable. Two experienced radiologists blinded to the clinical and laboratory data of the patients performed the studies.

The SPSS package (SPSS Inc., Chicago, IL, USA) was used to perform statistical analysis. Distribution of the groups was analyzed with one sample Kolmogrov-Smirnov test. The one-way ANOVA and Tukey *post hoc* analysis were performed to compare means among groups for normally distributed data. When the results were not normally distributed, nonparametric Kruskal-Wallis test was used and differences between groups were analyzed with Mann-Whitney U test. Receiver operating characteristic (ROC) curve was constructed to determine the best threshold value for AMH in predicting PCOS diagnosis. A P value < 0.05 was considered statistically significant.

## Results

The clinical characteristics and hormone profiles of patients are shown in Table 1. BMI, neck, waist, breast circumferences and waist hip ratio were similar among groups. A significant difference existed between the groups with regard to F-G scores, total testosterone and E2 levels (Table 2). Twelve patients in group 1 and eight patients in group 2 had high total testosterone levels. Biochemical markers of cardiovascular risks are shown in Table 2. Copeptin was not correlated with fasting insulin, TG, testosterone levels, CIMT, HOMA-IR, and FG score. It was positively correlated with BMI and serum cholesterol levels. Serum PTX3 was not statistically different in PCOS subjects. The mean serum fasting insulin levels and HOMA-IR levels were significantly higher in the adult PCOS group (Table 2).

**Table 1:** Descriptive Variables in Adolescent PCOS (n = 25), Adult PCOS (n = 25) and Control Subjects (n = 25)

Characteristics	Adolescent PCOS (n = 25)	Adult PCOS (n = 25)	Control (n = 25)	P value
Age (years)	17.96 ± 1.59^a^	27.24 ± 5.34^b^	31.8 ± 8.11^c^	0.001*
Body mass index (kg/m^2^)	23.75 ± 4.21	25.86 ± 4.61	25.44 ± 5.10	0.053
Waist circumference (cm)	91.82 ± 18.36	100 ± 20	123.55 ± 24.71	0.09
Hip circumference (cm)	98.28 ± 9.77^d^	106.52 ± 8.01	104.60 ± 10.27	0.007*
WHR	0.75 ± 0.15	0.77 ± 0.05	0.77 ± 0.07	0.97
FSH (mIU/mL)	5.74 ± 1.48	5.39 ± 1.79	5.61 ± 1.69	0.760
LH (mIU/mL)*	11.69 ± 6.78^e^	10.23 ± 6.36^f^	5.56 ± 2.60	0.001*
Total testosterone (ng/dL)	0.52 ± 0.20^g^	0.41 ± 0.22^h^	0.22 ± 0.11	0.001*
Estradiol (pg/mL)	50.53 ± 24.38^i^	48.48 ± 22.07^j^	74.84 ± 34.79	0.002*
M-Ferriman-Gallwey score	12.40 ± 3.37^k^	9.52 ± 3.38^l^	2.54 ± 1.43^m^	0.001*
Ovarian volume (mm^3^)	10.86 ± 3.76^n^	11.43 ± 4.55^o^	4.08 ± 2.15	0.001*

*Groups are significantly different from each other. ^a^Compared to group 2 (P = 0.001). ^b^Compared to group 3 (P = 0.001). ^c^Compared to group 1 (P = 0.015). ^d^Compared to group 2 (P = 0.008). ^e^Compared to group 3 (P = 0.001). ^f^Compared to group 3 (P = 0.01). ^g^Compared to group 3 (P = 0.001). ^h^Compared to group 3 (P = 0.002). ^i^Compared to group 3 (P = 0.0079). ^j^Compared to group 3 (P = 0.003). ^k^Compared to group 2 (P = 0.004). ^l^Compared to group 3 (P = 0.001). ^m^Compared to group 1 (P = 0.001). ^n^Compared to group 3 (P = 0.001). ^o^Compared to group 3 (P = 0.001). WHR: waist to hip ratio.

**Table 2:** Metabolic Profile of Adolescent PCOS (n = 25), Adult PCOS (n = 25) and Control Subjects (n = 25)

Characteristics	Adolescent PCOS (n = 25)	Adult PCOS (n = 25)	Control (n = 25)	P value
Fasting glucose (mg/dL)	86.80 ± 5.43	84.24 ± 17.73	89.84 ± 7.24	0.23
Fasting insulin (μIU/mL)	8.64 ± 4.07^a^	14.93 ± 7.38^b^	7.64 ± 4.07	0.001*
CRP (g/dL)	1.8 ± 0.43	2.01 ± 1.12	1.92 ± 0.32	0.54
HOMA-IR	1.63 ± 0.89^c^	3.33 ± 1.86^d^	1.70 ± 1.86	0.001*
TC (mg/dL)	177.20 ± 28.47	190.88 ± 45.62	164.58 ± 40.12	0.19
LDL (mg/dL)	99.20 ± 24.37	110.96 ± 41.73	99.36 ± 28.81	0.34
VLDL (mg/dL)	18.04 ± 7.67	24.39 ± 15.66	21.5 ± 10.13	0.32
HDL (mg/dL)	57.68 ± 12.14	56.52 ± 11.80	59.84 ± 16.71	0.91
TG (mg/dL)	91.20 ± 30.50	123.12 ± 42.81	93.56 ± 40.73	0.32
Pentraxin 3 (ng/mL)	1.36 ± 0.56	1.64 ± 0.72	2 ± 0.99	0.95
Copeptin (ng/mL)	0.001 ± 0.00^e^	0.28 ± 0.009	0.12 ± 0.00^f^	0.015*
amh (ng/mL)	9.72 ± 1.15^g^	7.9 ± 0.99^h^	3.52 ± 0.58	0.001*

Values are mean ± standard deviation. *Groups are significantly different from each other. ^a^Compared to group 2 (P = 0.001). ^b^Compared to group 3 (P = 0.001). ^c^Compared to group 2 (P = 0.001). ^d^Compared to group 3 (P = 0.001). ^e^Compared to group 2 (P = 0.005). ^f^Compared to group 1 (P = 0.077). ^g^Compared to group 3 (P = 0.001). ^h^Compared to group 3 (P = 0.001).

PCOS patients did not have a significantly higher CIMT (Table 3). All the studied cardiac parameters were not different among groups (Table 3).

**Table 3:** Common Carotid Artery Intima-Media Thickness and Cardiac Indices Among Groups

Characteristics	Adolescent PCOS (n = 25)	Adult PCOS (n = 25)	Control (n = 25)	P value
Carotid artery intima-media thickness (mm)	0.48 ± 0.06^a^	0.59 ± 0.06^b^	0.67 ± 0.13^c^	0.0001*
TA systolic (mm Hg)	105.60 ± 9^d^	114.80 ± 15.51	109.20 ± 8.62	0.021*
TA diastolic (mm Hg)	68.64 ± 6.65^e^	74.00 ± 8.53	72.00 ± 5.0^f^	0.018*
MPI	0.55 ± 0.09	0.55 ± 0.07	0.50 ± 0.08	0.09
Mass I (g/m^2^)	66.76 ± 14.29	109.65 ± 18.70	70.61 ± 11.91	0.45
Strain 100 (%)	12.66 ± 6.31	10.15 ± 4.75	13.24 ± 6.20	0.14
EP	3.75 ± 2.27	5.33 ± 3.86	3.69 ± 2.52	0.09
Stiffness	4.37 ± 2.65	5.56 ± 3.40	4.08 ± 2.58	0.17
Distensibility (cm^2^/dyn/10^3^ )	7.08 ± 3.78	5.30 ± 2.72	7.29 ± 3.43	0.07
E/ E’	6.63 ± 1.41	6.13 ± 1.33	6.43 ± 2.02	0.54

Values are mean ± standard deviation. *Groups are significantly different from each other. ^a^Compared to group 2 (P = 0.001). ^b^Compared to group 3 (P = 0.002). ^c^Compared to group 1 (P =0.001). ^d^Compared to group 2 (P = 0.016). ^e^Compared to group 2 (P = 0.012). ^f^Compared to group 1 (P = 0.029). MPI: myocard performance index; Mass I: left ventricular mass index; Strain: aortic strain; EP: elastic modulus; Stiffness: aortic stiffness index; E/E’: the ratio of peak early diastolic filling velocity of the mitral inflow to peak early diastolic velocity of the mitral annulus.

AMH levels were different among groups (Table 2). There was also a positive correlation between AMH and mean ovarian volume (r = 0.58, P < 0.001) and between AMH and total testosterone level (r = 0.63, P < 0.001).

In order to predict a threshold value for the diagnosis of PCOS by using AMH, the ROC method was used. Area under the curve was 0.820 and cut-off point was 6.66 for AMH with a sensitivity of 62% and specificity of 76%.

## Discussion

We aimed to investigate the role of possible markers in the prediction of PCOS diagnosis and PCOS-related metabolic alterations and cardiovascular events in adolescent subjects. Because of overlapping symptoms of pubertal development, diagnosis of PCOS in adolescence is difficult. All three definitions including National Institute of Health (NIH), Rotterdam and the Androgen Excess Society (AES) [8, 12, 13] for the diagnosis of PCOS were defined for adult population. Carmina et al proposed new criteria which all three of hyperandrogenism, chronic anovulation and polycystic ovaries must be present to make a definitive diagnosis of PCOS in adolescents [14]. In the present study we used diagnostic criteria recommended by Carmina et al for selecting the patients. It has been suggested that serum AMH levels were increased in PCOS [15]. Although AMH seems a promising diagnostic tool, Hart et al failed to demonstrate serum AMH was a reliable predictor of polycystic ovarian morphology or for the presence of PCOS in the general adolescent population [16]. Routine serum testing for AMH levels for the diagnosis of PCOS is controversial. In the present study, AMH levels among study groups were different. PCOS subjects had significantly higher AMH levels. There was a positive correlation between AMH and ovarian volume. AMH also positively correlated with total testosterone. We found AMH levels and testosterone high in adolescent PCOS group. In previous studies, AMH levels were found to be associated significantly with testosterone especially in adolescents [17]. This finding is harmonious with our results. We think that AMH can be a useful marker to determine the adolescent population who have a tendency to develop PCOS. But further studies are required with larger sample sizes. We also think that subgroups in adolescent PCOS group should be determined which are associated more significantly with elevated AMH levels in subsequent studies.

Previous studies have shown that the prevalence of metabolic syndrome in PCOS patients was higher [18]. In our study, there were no significant differences in serum concentrations of fasting glucose, CRP, serum lipids and waist to hip ratio among BMI similar PCOS and control subjects. The mean serum fasting insulin levels and HOMA-IR levels were significantly higher in the adult PCOS group. This finding was consistent with previous studies indicating that women with PCOS were hyperinsulinemic and insulin resistant, independent of obesity, compared with normal women [19]. Adolescent PCOS subjects did not have higher insulin levels or HOMA-IR levels in the present study. Possible explanation for this finding is that insulin resistance progressively deteriorates over time.

It was reported that elevated copeptin predicts increased risk for diabetes mellitus independently of fasting glucose and insulin [3]. Karbek et al analyzed copeptin in PCOS patients and found fasting insulin levels and HOMA-IR were higher in PCOS patients and copeptin level was positively correlated with fasting insulin, TG, free testosterone levels, CIMT, HOMA-IR, and F-G score [20]. In our study, copeptin was not correlated with fasting insulin, TG, testosterone levels, CIMT, HOMA-IR, and F-G score. It was positively correlated with BMI and serum cholesterol levels. Copeptin needs to be further investigated in obese PCOS patients.

PTX3 is an inflammatory marker and it is associated with cardiovascular disease risk factors [21]. The role of PTX3 in PCOS is so far inconclusive. Limited number of study has examined the relationship between PTX3 and PCOS. Tosi et al found that plasma PTX3 levels were reduced in women with PCOS [22]. Sari et al found PTX3 and CRP levels were similar in the PCOS compared to control group [23]. We found no difference in PTX3 and CRP concentrations between groups.

Ultrasound detection of the CIMT measurement is considered a surrogate marker of subclinical atherosclerosis [24]. According to a recent systematic review and meta-analysis women with PCOS demonstrated higher CIMT than control [25]. In the current study PCOS patients did not have a significantly higher CIMT. The prevalence of atherogenic CIMT pattern increases with age and BMI [26]. Detected differences in CIMT could be due to age differences among groups.

An increased risk of coronary heart disease in women with PCOS is inconclusive [27]. Our aim was to investigate whether the PCOS subjects had abnormal echocardiographic indices. We failed to demonstrate any abnormal echocardiographic indices in these patients. This may be due to study population who did not have glucose intolerance, dyslipidemia and high BMI.

One of the limitations of our study is relative small sample size and lack of obese PCOS control group. Another limitation was the existence of relatively older aged women in the control group: six women were older than 35 years old. However we think that this could also be an advantage in terms of comparing the metabolic alterations between these groups.

In conclusion, we failed to demonstrate the role of copeptin and PTX3 in PCOS. However we conclude that serum AMH may be a useful adjunct in the diagnosis of adolescent PCOS patients. Further studies with larger number of patients are recommended to investigate these possible markers in both lean and obese patients with PCOS.
